# Luteolin (LUT) Induces Apoptosis and Regulates Mitochondrial Membrane Potential to Inhibit Cell Growth in Human Cervical Epidermoid Carcinoma Cells (Ca Ski)

**DOI:** 10.3390/biomedicines12102330

**Published:** 2024-10-14

**Authors:** Sung-Nan Pei, Kuan-Ting Lee, Kun-Ming Rau, Tsung-Ying Lin, Tai-Hsin Tsai, Yi-Chiang Hsu

**Affiliations:** 1 Department of Hematology Oncology, E-Da Cancer Hospital, I-Shou University, Kaohsiung 82445, Taiwan; sungnanpei@gmail.com (S.-N.P.); liu07822@ms57.hinet.net (K.-M.R.); 2 Division of Neurosurgery, Department of Surgery, Kaohsiung Municipal Ta-Tung Hospital, Kaohsiung 80145, Taiwan; ayta860404@gmail.com (K.-T.L.); tsaitaihsin@gmail.com (T.-Y.L.); 3 Graduate Institute of Medicine, College of Medicine, Kaohsiung Medical University, Kaohsiung 807378, Taiwan; 4 School of Medicine, I-Shou University, Kaohsiung 82445, Taiwan; 5 Division of Neurosurgery, Department of Surgery, Kaohsiung Medical University Hospital, Kaohsiung 807378, Taiwan; 6 Department of Surgery, School of Medicine, College of Medicine, Kaohsiung Medical University, Kaohsiung 807378, Taiwan

**Keywords:** luteolin, cervical epidermoid cancer, apoptosis, mitochondrial, caspase activation

## Abstract

*Background/Objectives:* Luteolin (LUT) is a natural flavonoid with known anti-inflammatory, antioxidant, and anti-cancer properties. Cervical cancer, particularly prevalent in certain regions, remains a significant health challenge due to its high recurrence and poor response to treatment. This study aimed to investigate the anti-tumor effects of LUT on human cervical epidermoid carcinoma cells (Ca Ski), focusing on cell growth inhibition, apoptosis induction, and regulation of mitochondrial membrane potential. *Methods:* Ca Ski cells were treated with varying concentrations of LUT (0, 25, 50, 100 µM) for different time periods (24, 48, 72 hours). Cell viability was measured using the MTT assay, apoptosis was assessed by flow cytometry with annexin V-FITC/PI staining, and changes in mitochondrial membrane potential were evaluated using JC-1 staining. Caspase-3 activation was examined by flow cytometry, and expression of apoptosis-related proteins (caspase-3, -8, -9, AIF) was analyzed via Western blotting. *Results:* LUT significantly inhibited the growth of Ca Ski cells in a dose- and time-dependent manner, with the most pronounced effects observed at 100 µM over 72 hours. Flow cytometry confirmed that LUT induced apoptosis without causing necrosis. Mitochondrial membrane potential was reduced after LUT treatment, coinciding with increased caspase-3 activation. Western blot analysis revealed the upregulation of pro-apoptotic proteins caspase-3, -8, -9, and AIF, indicating that LUT induces apoptosis through the intrinsic mitochondrial pathway. *Conclusions:* Luteolin effectively inhibits cervical cancer cell proliferation and induces apoptosis by disrupting mitochondrial membrane potential and activating caspases. These findings suggest that LUT holds potential as a therapeutic agent for cervical cancer, with further studies needed to explore its in vivo efficacy and broader clinical applications.

## 1. Introduction

Luteolin (LUT) is a natural flavonoid found in various plants, including fruits, vegetables, and herbs [1]. It has been extensively studied for its potential health benefits and has been shown to exhibit a wide range of pharmacological activities, including anti-inflammatory, antioxidant, and anticancer properties [2,3]. Research indicates that LUT can inhibit the growth and proliferation of cancer cells in various types of cancer [4,5,6,7]. Additionally, it has been shown to induce apoptosis, or programmed cell death, in cancer cells [8] and inhibit cancer cell invasion and migration [9]. LUT also exhibits neuroprotective, cardioprotective, and anti-diabetic effects, suggesting its potential in preventing and treating various chronic diseases [10]. The versatility and potential health benefits of LUT make it an attractive compound for further research and development.

Cervical cancer is a significant health concern for women worldwide and is particularly prevalent in Taiwan, where it is the most common malignancy among women and a leading cause of death from gynecological cancers [11]. Most women diagnosed with this cancer exhibit widely disseminated tumors and poor survival rates [12]. Research suggests human papillomavirus (HPV) infection, genetic susceptibility, and various other factors play an important role in the development of cervical cancer. Relevant evidence shows that the carcinogenic types of HPV play many important roles in the occurrence and development of precancerous lesions of cervical cancer [13]. Numerous studies indicate that the effects of natural plant components are positively correlated with a reduced incidence of certain tumors, including ovarian, cervical, and lung cancers [13,14,15]. 

Previous studies have confirmed that LUT has an inhibitory effect on the proliferation of cancer cells [16]. However, whether LUT inhibits the growth of cervical cancer cells remains unclear. Furthermore, the mechanism of LUT’s anticancer effects has not yet been determined. The objective of this study is to investigate whether LUT contributes to the anti-proliferative effects and the induction of apoptosis in cervical cancer cells (Ca Ski). It is anticipated that these experiments will provide medical support and scientific and technological assistance for the further development of cervical cancer treatments.

## 2. Materials and Methods

### 2.1. Materials

Luteolin (LUT), with a purity greater than 99%, was purchased from Sigma (St. Louis, MO, USA). It was dissolved in dimethyl sulfoxide (DMSO), aliquoted, and stored at −20 °C. DMSO and MTT [3-(4,5-dimethylthiazol-2-yl)-2,5-diphenyltetrazolium bromide] were also obtained from Sigma (St. Louis, MO, USA). Cell culture medium (RPMI 1640), phosphate-buffered saline (PBS), antibiotics, sodium pyruvate, L-glutamine, trypsin, and fetal bovine serum (FBS) were sourced from Gibco BRL (Grand Island, NY, USA). Polyvinylidene fluoride (PVDF) membranes and molecular weight markers were purchased from Bio-Rad (Laboratories Inc., Taiwan Branch, Taipei, Taiwan). All other reagents and compounds used were of analytical grade.

### 2.2. Cells

The Ca Ski cells, a human cervical epidermoid carcinoma cell line derived from a metastatic site in the small intestine, were purchased from ATCC. These cells were maintained in culture dishes with 90% (*v*/*v*) RPMI 1640 medium supplemented with 2 mM L-glutamine, Earle’s Balanced Salt Solution (BSS) adjusted to contain 1.5 g/L sodium bicarbonate, 0.1 mM non-essential amino acids (NEAA), 1 mM sodium pyruvate, and 10% (*v*/*v*) fetal bovine serum (FBS). They were cultured in a 37 °C incubator with an atmosphere of 5% CO_2_.

### 2.3. Cell Proliferation Assay

Cancer cells were seeded into a 96-well culture plate at a density of 5000 cells per well. The cells were treated with varying concentrations of luteolin (LUT) (0, 25, 50, and 100 µM), with LUT forming a complex with the culture medium. The cells were then incubated at 37 °C for 24, 48, and 72 h in a CO_2_ incubator. After the respective incubation periods, MTT dye (1 mg/mL) was added to each well and incubated for at least 4 h. The reaction was terminated by the addition of dimethyl sulfoxide (DMSO), and optical density was measured at OD540 using a multi-well plate reader. Background absorbance from the medium without cells was subtracted. All samples were assessed in triplicate, and the mean for each experiment was calculated. Results were expressed as a percentage of the control, which was set at 100%. Each assay was performed in triplicate, and results were presented as the mean ± SEM.

### 2.4. Measurement of Apoptosis/Necrosis

The Ca Ski cells were initially seeded in 6-well plates (Orange Scientific, E.U., Braine-l’Alleud, Belgium). After treating the cells with luteolin (LUT) for 4 h, they were harvested and centrifuged again, discarding the supernatant. The cells were then resuspended and incubated in 1× annexin-binding buffer. Following this, 5 μL of annexin V-FITC (BD Pharmingen, BD, Franklin Lakes, NJ, USA) and 5 μL of a 100 μg/mL propidium iodide (PI) working solution was added, and the cells were incubated for 15 min. After incubation, the stained cells were analyzed using flow cytometry (FACSCalibur, BD Pharmingen, USA). Data analysis was performed using WinMDI 2.9 software (BD, USA).

### 2.5. Caspase 3 Activity Assay

Caspase activity was assessed using FITC-conjugated rabbit anti-active caspase 3 antibody (BD Pharmingen, USA). The cells were treated with luteolin (LUT) for 24 h. After treatment, caspase activity was detected and analyzed by flow cytometry (FACSCalibur, BD, USA). Data analysis was performed using WinMDI 2.9 software (BD, USA).

### 2.6. Mitochondrial Membrane Potential (MMP) 

The cell lines were initially seeded in 6-well plates (Orange Scientific, E.U.). After treating the cells with luteolin (LUT) for 4 h, JC-1 dye (25 µM) was added to the culture medium (500 µL per well) and incubated at 37 °C for 20 min to stain the mitochondria. Following incubation, the cells were washed twice with warm PBS and then fixed with 2% paraformaldehyde. For the quantification of JC-1, flow cytometry was performed using a BD FACScalibur (BD, USA). Mitochondria containing red JC-1 aggregates in healthy cells were detected in the FL2 channel, while green JC-1 monomers in apoptotic cells were detected in the FL1 channel.

### 2.7. Cell Cycle Analysis 

For cell cycle analysis, propidium iodide (PI), a fluorescent nucleic acid dye, was used to determine the proportion of cells in each phase of the cell cycle. Cells were treated with luteolin (LUT) for 24 h, then harvested and fixed in 1 mL of cold 75% ethanol overnight at −20 °C. Following fixation, DNA was stained with a PI/RNase A solution, and the DNA content was analyzed using flow cytometry. Data analysis was conducted with WinMDI 2.9 software.

### 2.8. Western Blot Assay 

A total of 30–50 µg of protein samples was separated by 10–12% SDS-PAGE and transferred to PVDF membranes (Millipore, Burlington, MA, USA). The membranes were blocked overnight with blocking buffer (Odyssey, LI-COR, Lincoln, NE, USA) and then incubated for 1.5–2 h with the following primary antibodies: anti-β-actin (Sigma-Aldrich, St. Louis, MO, USA), anti-caspase 3 (sc-7148), anti-caspase 8 (sc-6134), anti-caspase 9 (sc-7885), and anti-AIF (sc-9417) (Santa Cruz Biotechnology, Santa Cruz, CA, USA). After washing, the membranes were incubated with a secondary antibody conjugated to horseradish peroxidase (HRP) at a 1:20,000 dilution for 30 min. Protein–antibody complexes were then visualized using a chemiluminescence detection kit (ECL; Amersham Corp., Arlington Heights, IL, USA).

### 2.9. Statistical Analysis

All data are presented as the mean ± SEM from at least three independent experiments. Statistical analysis was performed using a *t*-test or one-way ANOVA with post hoc tests, and significance was defined as *p* < 0.05.

## 3. Results

### 3.1. LUT Inhibited the Cell Growth of Ca Ski Cells by MTT Assay

**Figure 1:**
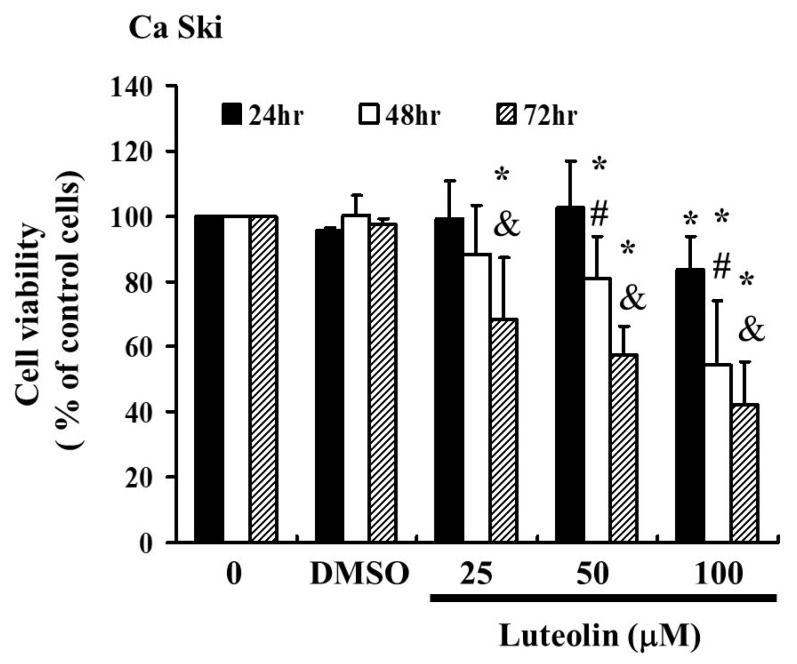
LUT affects the survival of cervical cancer cells (Ca Ski) and inhibits their proliferation. Ca Ski cells were treated with varying concentrations of LUT (0, 25, 50, and 100 µM) for 24 to 72 h, and cell viability was measured using the MTT assay. Results are presented as a percentage relative to the control, which is set at 100%. Data are expressed as the mean ± SEM from at least three experiments. Statistical significance was determined using a *t*-test, with * *p* < 0.05 indicating a significant difference from the control group, # *p* < 0.05 indicating a significant difference from the 24 h group, and & *p* < 0.05 indicating a significant difference from the 48 hrs group.

To assess whether luteolin (LUT) could effectively inhibit the activity of cervical cancer cells, Ca Ski cells were treated with LUT at concentrations of 0, 25, 50, and 100 µM for 24 to 72 h. The MTT assay results, shown in Figure 1 indicate that LUT significantly suppressed the proliferation of Ca Ski cells compared to the control group. The data demonstrate that LUT treatment reduced cell survival and proliferation in a dose- and time-dependent manner, with the following relationships observed: y = −2.5498x + 103.86y = −2.5498x + 103.86y = −2.5498x + 103.86 (R^2^ = 0.2943) at 24 h, y = −11.044x + 117.85y = −11.044x + 117.85y = −11.044x + 117.85 (R^2^ = 0.8596) at 48 h, and y = −15.602x + 119.91y = −15.602x + 119.91y = −15.602x + 119.91 (R^2^ = 0.9526) at 72 h. However, LUTs have also been identified at various concentrations (0–100 μM). This is consistent with the experimental results [17].

### 3.2. LUT Induced Apoptosis but Not Necrosis in Ca Ski Cells

**Figure 2:**
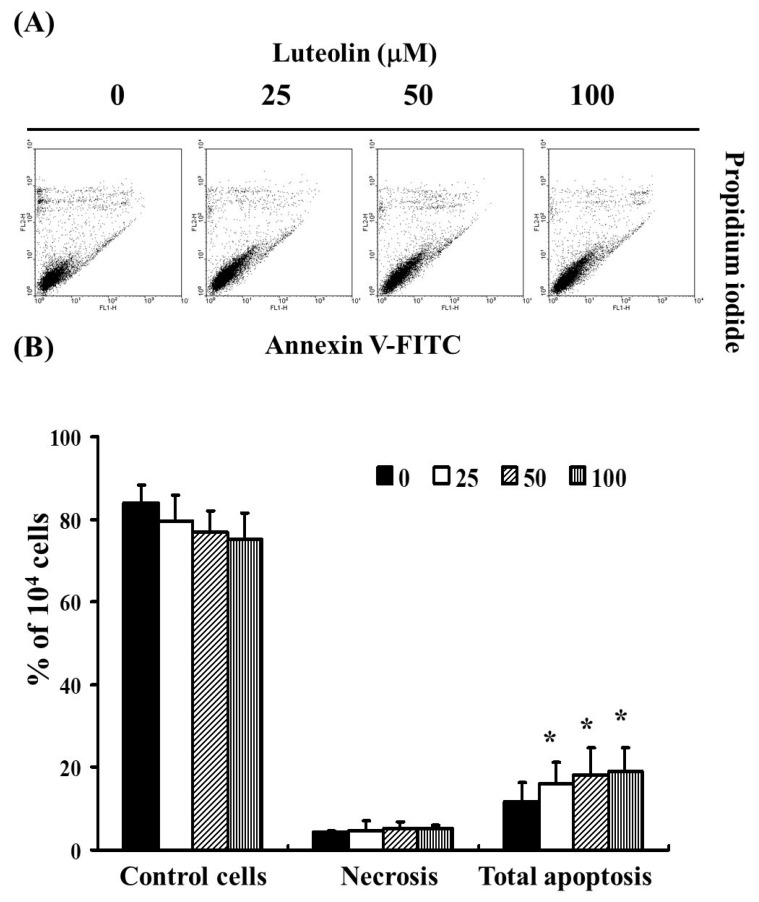
The role of LUT in inducing cell apoptosis in cervical cancer cells (Ca Ski). (**A**) The extent of total apoptosis and necrosis in Ca Ski cells after 4 h of incubation with LUT. (**B**) Results are presented as a percentage relative to the control group, including necrosis and the total number of apoptotic cells (both early and late apoptosis). Statistical significance was assessed using a *t*-test, with * *p* < 0.05 indicating a significant difference compared to the control group.

To assess the effect of LUT on cell apoptosis and necrosis in cervical cancer cells, we analyzed Ca Ski cells exposed to LUT at concentrations of 0, 25, 50, and 100 µM for 4 h. Cells were then stained with Annexin V-FITC and propidium iodide (PI) to detect apoptotic cell formation. As shown in Figure 2A, flow cytometry analysis was used to determine the percentage of total apoptotic cells, including both early and late apoptotic stages. The results indicated that LUT treatment significantly increased the percentage of apoptotic cells without affecting necrosis (Figure 2B). These findings suggest that LUT induces cell apoptosis but does not affect necrosis in Ca Ski cells.

### 3.3. LUT Induced Accumulation of Cell Cycle Sub-G1 Phase in Ca Ski Cells

**Figure 3:**
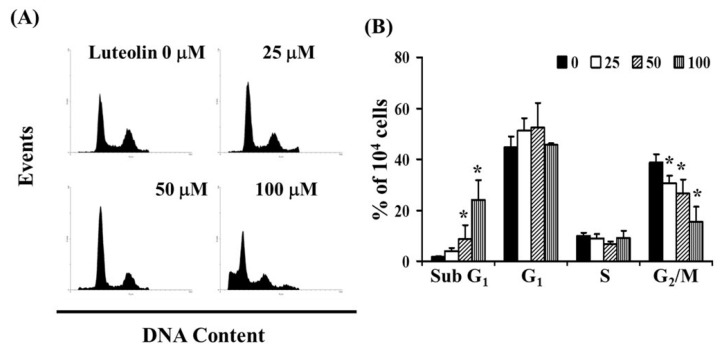
Influence of LUT on cell cycle progression/distribution in cervical cancer cells. (**A**) Cell cycle analysis of Ca Ski cells after 24 h of treatment with LUT. (**B**) LUT treatment led to an increase in the percentage of cells in the sub-G1 phase. Results are presented as the mean ± SD from three experiments. The asterisk (*) in each bar group indicates that the difference compared to the 0 µM LUT treatment is statistically significant at *p* < 0.05.

To investigate whether LUT affects the cell cycle, we performed flow cytometry analysis on Ca Ski cells exposed to LUT (0, 25, 50, and 100 µM) for 24 h. As shown in Figure 3A, LUT treatment led to a reduction in the number of cells in the G2/M phase and an increase in the sub-G1 phase, which may indicate apoptosis in cervical cancer cells. The data revealed that LUT treatment increased the sub-G1 cell population (y = 0.2303x − 0.4767, R^2^ = 0.949) while decreasing the number of cells in the G2/M phase (y = −0.2269x + 37.744, R^2^ = 0.9892) compared to the control (* *p* < 0.05 vs. 0 µM LUT) (Figure 3B).

### 3.4. LUT-Induced Apoptosis Could Be Related to Changes in Mitochondrial Membrane Potential

**Figure 4:**
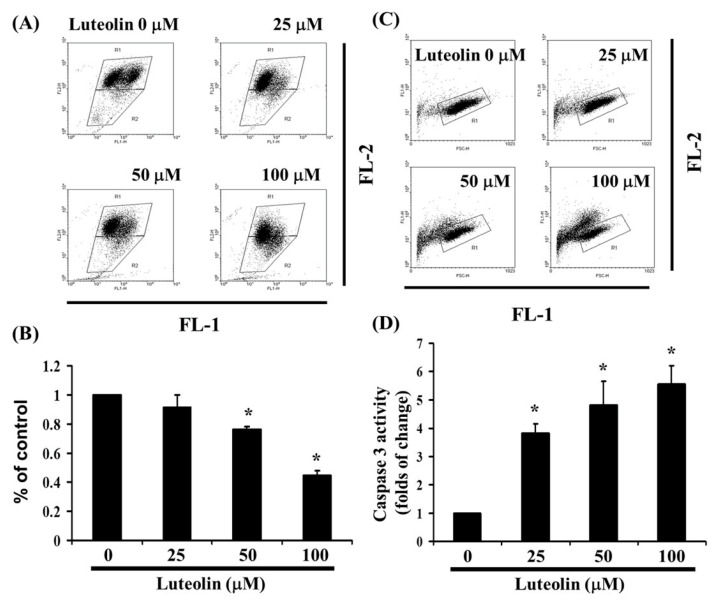
The effect of LUT on caspase 3 activation and mitochondrial membrane potential. (**A**) LUT-induced changes in mitochondrial membrane potential (ΔΨm) were assessed in Ca Ski cells. ΔΨm levels were determined using JC-1 staining and flow cytometry. (**B**) Cells were treated with varying concentrations of LUT for 24 h. Statistical significance is indicated by * *p* < 0.05. (**C**) Following 24 h treatment with LUT, cells were harvested and labeled with FITC-conjugated anti-active caspase 3 antibody. (**D**) Caspase 3 activation was quantified by flow cytometry. All data are expressed as mean ± SD from three independent experiments, with statistical significance denoted by * *p* < 0.05.

As shown in Figure 2, LUT induces cell apoptosis. To investigate the loss of mitochondrial membrane potential, a hallmark of early apoptosis that coincides with caspase activation, we used JC-1 staining. In non-apoptotic cells, JC-1 exists as red-fluorescent aggregates within the mitochondria and as green-fluorescent monomers in the cytosol. In apoptotic and necrotic cells, JC-1 remains in its monomeric form, resulting in green fluorescence throughout the cytosol. Ca Ski cells were exposed to LUT (0, 25, 50, and 100 µM) for 4 h. JC-1 staining revealed that LUT treatment decreased mitochondrial membrane potential, as indicated by the reduction of red-fluorescent J-aggregates in FL-1/FL-2 dot plots (Figure 4A,B). These results suggest that LUT significantly reduces the mitochondrial membrane potential of Ca Ski cells (y = −0.1817x + 1.2338, R^2^ = 0.9245). To determine if LUT-induced apoptosis affects caspase-3 activity, we treated Ca Ski cells with LUT (0, 25, 50, and 100 µM) and stained them with anti-active caspase 3 antibodies for flow cytometry analysis. As shown in Figure 4C,D, caspase 3 activity was significantly increased with LUT treatment (y = 1.4678x + 0.1345, R^2^ = 0.8982).

### 3.5. Effect of LUT-Induced Apoptosis Associated with Caspase 3, 8, and 9 Expression

**Figure 5:**
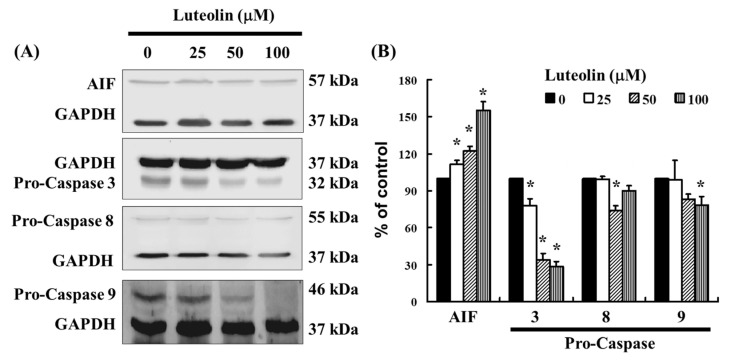
The expression of LUT-regulated caspase family in Ca Ski cells. (**A**) The expression of pro-caspase 3, 8, 9, and AIF were assessed by Western blot analysis in Ca Ski cells. (**B**) The quantification of pro-caspase 3, 8, 9, and AIF is shown in the bar graph. Data were normalized to GAPDH as a control and expressed as a percentage relative to the control group, which was set at 100%. All results are presented as the mean (±SEM) of at least three independent experiments. Statistical significance was determined using a *t*-test, with * *p* < 0.05 indicating significant differences compared to the control group.

As shown in Figure 3, LUT induces caspase 3 activation in Ca Ski cells, as demonstrated by flow cytometry. To further investigate the effect of LUT on apoptosis, we examined the expression of apoptosis-related proteins using Western blot analysis. The results revealed that LUT treatment significantly altered the expression levels of pro-caspase 3, 8, and 9 (Figure 5A,B). Additionally, LUT increased the expression of AIF, indicating its involvement in apoptosis induction (Figure 5B). Collectively, these findings suggest that LUT may inhibit the growth of cervical cancer cell lines by promoting apoptotic activation.

## 4. Discussion

LUT is a well-known phosphodiesterase inhibitor with documented effects on cholesterol synthesis and a range of biological activities, including anticancer, anti-diabetic, antioxidant, anti-inflammatory, anti-atherosclerotic, and antiviral properties [16,18]. It has long been utilized in traditional Asian medicine for treating conditions related to oxidative stress and acute inflammation, such as endotoxemia, acute lung injury, acute myocardial infarction, and hepatitis [19]. Studies have also indicated that LUT may contribute to insulin resistance in mice [20]. LUT has hydroxyl groups at the 3′, 4′, 5, and 7 positions of its flavonoid structure. These hydroxyl groups contribute significantly to its antioxidant activity by scavenging free radicals and neutralizing reactive oxygen species (ROS) [1,21]. ROS generation is often involved in cancer cell survival, and the presence of these hydroxyl groups enhances LUT’s ability to protect cells from oxidative stress. In the context of cancer, this property can support the induction of apoptosis by increasing oxidative damage in cancer cells [8,22,23]. The hydroxyl groups contribute to luteolin’s ability to modulate signaling pathways, particularly the inhibition of pro-survival pathways and the activation of apoptosis pathways such as caspases [24], which also corroborates our experimental data on activating caspase 3, as shown in Figure 4 and Figure 5.

Recently, LUT has been identified as an effective inducer of heme oxygenase-1 (HO-1), leading to the reduced production of nitric oxide (NO) by inducible nitric oxide synthase and the suppression of nuclear factor-κB (NF-κB) pathway activation in inflammatory diseases [23]. These properties suggest that LUT may offer a promising strategy for cancer prevention and treatment by inhibiting cancer cell growth and inducing apoptosis. In recent years, natural medicines, particularly flavonoid compounds, have gained attention for their chemoprotective functions, showing potential in reducing cardiovascular disease risk and cancer [25,26,27]. 

In both in vitro and in vivo studies, LUT effectively disrupts cell cycle progression at multiple phases, including G0/G1, S, and G2/M. This disruption, coupled with its capacity to induce apoptosis, serves as a significant barrier to cancer cell advancement [28]. LUT has been shown to increase apoptosis and induce cell cycle arrest at the G2 phase in colorectal cancer (CRC) cells, corroborating previous findings [29]. Exposure to luteolin in cancer cells results in DNA damage, primarily in the form of single-strand breaks, which is a crucial factor in the induction of apoptosis [30]. Our findings with Ca Ski cells treated with luteolin align with these observations, demonstrating the induction of the sub-G1 phase and the activation of apoptosis, as shown in Figure 2 and Figure 3. 

In previous reports, other compounds besides LUT, such as apigenin, can induce apoptosis via mitochondrial pathways and caspase activation. Apigenin has been demonstrated to induce G2/M phase arrest and apoptosis through downregulating anti-apoptotic proteins like Bcl-2, similar to LUT [27,31]. However, curcumin and LUT also exert their anticancer effects by modulating oxidative stress and ROS production, as well as inhibiting key pro-survival signaling pathways, such as NF-κB and PI3K/Akt. Both compounds are also known to induce cell cycle arrest and apoptosis through caspase activation [31,32,33].

In this study, LUT exhibited significant anti-proliferative activity and the ability to induce apoptosis. Our data show that different concentrations of luteolin tested for 24, 48, and 72 h showed dose- and time-dependent effects, as shown in Figure 1. The results demonstrate this relationship, and we can further explore how higher or lower doses or longer treatment durations affect suppression. For example, cells may become resistant to LUTs over time. The experimental data support the conclusion that LUT can effectively arrest the growth of cervical cancer cells. Mechanistic analysis reveals that both the inhibition of cell proliferation and the induction of apoptosis are strongly dependent on the accumulation of LUT within the cancer cells, providing a broader context for clinical applications.

## 5. Conclusions

In conclusion, this study provides the first evidence that LUT effectively inhibits cervical epidermoid carcinoma. The data highlight LUT’s role in suppressing tumor growth through the induction of apoptosis. These findings suggest that LUT has potential as an anti-tumor agent for the chemoprevention of cervical cancer.

## Data Availability

The data underlying this article are available from the corresponding author on reasonable request.

## References

[1] Lo S., Leung E., Fedrizzi B., Barker D. (2021). Syntheses of mono-acylated luteolin derivatives, evaluation of their antiproliferative and radical scavenging activities and implications on their oral bioavailability. Sci. Rep..

[2] Hussain Y., Cui J.H., Khan H., Aschner M., Batiha G.E.-S., Jeandet P. (2021). Luteolin and cancer metastasis suppression: Focus on the role of epithelial to mesenchymal transition. Med. Oncol..

[3] Theoharides T.C. (2021). Luteolin: The wonder flavonoid. BioFactors.

[4] Ahmed S., Khan H., Fratantonio D., Hasan M.M., Sharifi S., Fathi N., Ullah H., Rastrelli L. (2019). Apoptosis induced by luteolin in breast cancer: Mechanistic and therapeutic perspectives. Phytomedicine.

[5] Naiki-Ito A., Naiki T., Kato H., Iida K., Etani T., Nagayasu Y., Suzuki S., Yamashita Y., Inaguma S., Onishi M. (2020). Recruitment of miR-8080 by luteolin inhibits androgen receptor splice variant 7 expression in castration-resistant prostate cancer. Carcinogenesis.

[6] Zhang M., Wang R., Tian J., Song M., Zhao R., Liu K., Zhu F., Shim J., Dong Z., Lee M. (2021). Targeting LIMK1 with luteolin inhibits the growth of lung cancer in vitro and in vivo. J. Cell Mol. Med..

[7] Song Y., Yu J., Li L., Wang L., Dong L., Xi G., Lu Y.J., Li Z. (2022). Luteolin impacts deoxyribonucleic acid repair by modulating the mitogen-activated protein kinase pathway in colorectal cancer. Bioengineered.

[8] Chen P., Zhang J.Y., Sha B.B., Ma Y.E., Hu T., Ma Y.C., Sun H., Shi J.X., Dong Z.M., Li P. (2017). Luteolin inhibits cell proliferation and induces cell apoptosis via down-regulation of mitochondrial membrane potential in esophageal carcinoma cells EC1 and KYSE450. Oncotarget.

[9] Yao Y., Rao C., Zheng G., Wang S. (2019). Luteolin suppresses colorectal cancer cell metastasis via regulation of the miR-384/pleiotrophin axis. Oncol. Rep..

[10] Huang L., Kim M.Y., Cho J.Y. (2023). Immunopharmacological Activities of Luteolin in Chronic Diseases. Int. J. Mol. Sci..

[11] Wu C.J., Chang W.C., Chen C.H., Huang S.C., Sheu B.C. (2017). Radical trachelectomy for early stage cervical cancer: A case series and literature review. Taiwan J. Obstet. Gynecol..

[12] Castle P.E., Pierz A. (2019). (At Least) Once in Her Lifetime: Global Cervical Cancer Prevention. Obstet. Gynecol. Clin. N. Am..

[13] Dillner J. (2019). Early detection and prevention. Mol. Oncol..

[14] Pellizzon A.C.A. (2018). Pain relief procedures before high-dose-rate brachytherapy for non-surgical treatment of cervix cancer. J. Contemp. Brachytherapy.

[15] Imran M., Rauf A., Shah Z.A., Saeed F., Imran A., Arshad M.U., Ahmad B., Bawazeer S., Atif M., Peters D.G. (2019). Chemo-preventive and therapeutic effect of the dietary flavonoid kaempferol: A comprehensive review. Phytother. Res..

[16] Tuorkey M.J. (2016). Molecular targets of Luteolin in cancer. Eur. J. Cancer Prev..

[17] Chen Y.-H., Wu J.-X., Yang S.-F., Hsiao Y.-H. (2023). Synergistic Combination of Luteolin and Asiatic Acid on Cervical Cancer In Vitro and In Vivo. Cancers.

[18] Wang L., Martins-Green M. (2014). Pomegranate and its components as alternative treatment for prostate cancer. Int. J. Mol. Sci..

[19] Kritas S.K., Saggini A., Varvara G., Murmura G., Caraffa A., Antinolfi P., Toniato E., Pantalone A., Neri G., Frydas S. (2013). Luteolin inhibits mast cell-mediated allergic inflammation. J. Biol. Regul. Homeost. Agents.

[20] Weng C.J., Yen G.C. (2012). Flavonoids, a ubiquitous dietary phenolic subclass, exert extensive in vitro anti-invasive and in vivo anti-metastatic activities. Cancer Metastasis Rev..

[21] Chen S., Wang X., Cheng Y., Gao H., Chen X. (2023). A Review of Classification, Biosynthesis, Biological Activities and Potential Applications of Flavonoids. Molecules.

[22] Nabavi S.F., Nabavi S.M., Mirzaei M., Moghaddam A.H. (2015). Luteolin as an anti-inflammatory and neuroprotective agent: A brief review. Brain Res. Bull..

[23] Zhou Q., Wang W. (2017). Luteolin induces apoptosis in HCT116 cells through reactive oxygen species (ROS)-mediated endoplasmic reticulum stress. Cell Stress Chaperones.

[24] Parcheta M., Świsłocka R., Orzechowska S., Akimowicz M., Choińska R., Lewandowski W. (2021). Recent Developments in Effective Antioxidants: The Structure and Antioxidant Properties. Materials.

[25] Zhao L., Lee J.Y., Hwang D.H. (2011). Inhibition of pattern recognition receptor-mediated inflammation by bioactive phytochemicals. Nutr. Rev..

[26] López-Lázaro M. (2009). Distribution and biological activities of the flavonoid Luteolin. Mini Rev. Med. Chem..

[27] Lin Y., Shi R., Wang X., Shen H.M. (2008). Luteolin, a flavonoid with potential for cancer prevention and therapy. Curr. Cancer Drug Targets.

[28] Seelinger G., Merfort I., Wölfle U., Schempp C.M. (2008). Anti-carcinogenic effects of the flavonoid Luteolin. Molecules.

[29] Krifa M., Leloup L., Ghedira K., Mousli M., Chekir-Ghedira L. (2014). Luteolin induces apoptosis in BE colorectal cancer cells by downregulating calpain, UHRF1, and DNMT1 expressions. Nutr. Cancer.

[30] Leung H.W., Wu C.H., Lin C.H., Lee H.Z. (2005). Luteolininduced DNA damage leading to human lung squamous carcinoma CH27 cell apoptosis. Eur. J. Pharmacol..

[31] Cao X., Liu X., Wang Y. (2016). Luteolin and apigenin decrease the secretion of pro-inflammatory cytokines and alter microRNA expression in LPS-stimulated RAW264.7 cells. Mol. Cell. Biochem..

[32] Hu P., Li K., Peng X.-X., Kan Y., Yao T.-J., Wang Z.-Y., Li Z., Liu H.-Y., Cai D. (2023). Curcumin derived from medicinal homologous foods: Its main signals in immunoregulation of oxidative stress, inflammation, and apoptosis. Front. Immunol..

[33] Wang T., Wu X., Al rudaisat M., Song Y., Cheng H. (2020). Curcumin induces G2/M arrest and triggers autophagy, ROS generation and cell senescence in cervical cancer cells. J. Cancer.

